# The Ingroup–Outgroup Relationship Influences Their Humanity: A Moderation Analysis of Status and Gender

**DOI:** 10.3389/fpsyg.2021.725898

**Published:** 2021-11-29

**Authors:** Matías Arriagada-Venegas, David Pérez-Jorge, Eva Ariño-Mateo

**Affiliations:** ^1^Department of Psychology, University of Concepción, Concepción, Chile; ^2^Department of Didactics and Educational Research, University of La Laguna, San Cristóbal de La Laguna, Spain; ^3^Department of Psychology, European University, Valencia, Spain

**Keywords:** organizational dehumanization, gender, status, teacher–student relationship, moderation

## Abstract

The aim of this study is to examine whether gender and status moderate the teacher–student relationship (TSR) and the perception of dehumanization in teachers and students. A total of 528 participants from a university in Laguna (74% students and 26% professors) completed a questionnaire based on the TSR scale, organizational dehumanization, and demographic variables. PROCESS, a mediation and moderation package, was used to analyze data. The results indicated that ingroup–outgroup relationship significantly influences the perception of organizational dehumanization (*p* < 0.001). In addition, gender (*p* < 0.001) and status (*p* < 0.001) have moderating roles. Specifically, female students are at most risk of perceiving themselves dehumanized, and males with high status (teachers) are less vulnerable to dehumanization. These findings are highly significant for the advancement of knowledge of the intergroup relationship and organizational dehumanization and have practical implications for teachers and students.

## Introduction

The theory of dehumanization was recently introduced in the organizational context (Christoff, 2014; Caesens et al., 2017) and is defined as employees’ perception of feeling like objects or machines (Haslam, 2006). Dehumanization can be mechanistic and animalistic, and this has been referred to as the “dual of dehumanization” theory. Mechanistic dehumanization involves the denial of attributes that differentiate humans from inert objects, e.g., interpersonal warmth and emotional responsiveness. This form is especially relevant in contexts such as technology and medicine. Second, animalistic dehumanization refers to the denial of characteristics that distinguish humans from animals. This form is mainly mentioned in contexts of ethnicity, immigration, and war (Nguyen et al., 2021).

Studies have investigated the effects that cause a high/low perception of organizational dehumanization and its relationship with other organizational variables such as job satisfaction, turnover, psychosomatic tension, authentic leadership, organizational citizenship behavior, and emotional exhaustion (Caesens et al., 2019; Sarwar, 2020; Arriagada-Venegas et al., 2021). These studies found that employees who perceived themselves as dehumanized had lower levels of organizational citizenship behavior, and job satisfaction, and higher levels of turnover intentions. However, how organizational dehumanization affects educational settings, such as the university context, and how the teacher–student relationship (TSR) influences Students’ perceptions of dehumanizations, is still is unknown. The significance of this study is that it examines how the relationship between ingroup (students) and outgroup (teachers) influences Students’ organizational dehumanization. In addition, two moderating variables, status and gender, are analyzed. The aim of this study is to analyze how the relationship with the outgroup influences ingroup dehumanization and how outgroup status (considering that teachers are high status and students are low status) and ingroup gender affect ingroup dehumanization.

Organizational dehumanization analyzes the denial or lesser attribution of humanity toward workers or leaders of organizations, who can be considered or metaphorized as objects. Dehumanization in organizations is a negative experience that affects the individual and is likely to dissociate from the organization (Bell and Khoury, 2011). Christoff (2014) states that dehumanization in the organization can harm the well-being of workers, as it increases their level of anxiety or depression. In the same vein, Baldissarri et al. (2014) found that workers who felt perceived as an instrument by their supervisor reported higher levels of burnout. Andrighetto et al. (2017) show that several key characteristics of work, such as repetition of movements, fragmentation of activities, and dependency, increase the perception of mechanistic dehumanization in workers. In addition, research results Bell and Khoury’s (2016) showed that organizational justice reduced workers’ perceptions of dehumanization by satisfying the principles of equality and treatment.

When people are mechanically dehumanized, they are considered as objects, as beings lacking the capacity to feel. These people enter states of “cognitive deconstructive” characterized by diminished clarity of thought, emotional numbness, and cognitive inflexibility. The experience of this type of dehumanization leads to pervasive emotions of sadness, anger, guilt, and shame (Bastian and Haslam, 2011). Furthermore, when a person is dehumanized, their status is reduced and attitudes of condescension and degradation are maintained by perceiving them as incompetent and unsophisticated (Vohs et al., 2007).

According to the TSR perspective, teachers evidence important ethical principles and virtues when they obtain and generate pleasant, caring, and understanding relationships toward the student and also demonstrate fairness, compassion, and understanding (Campbell, 2003). In addition, close and supportive relationships, characterized by open communication, trust, and responsiveness, provide students with emotional security to cope more effectively with academic and social stressors and to perceive a sense of belonging in the classroom context (Bosman et al., 2018; Hughes and Cao, 2018). Applying this perspective, researchers have assessed mainly two dimensions of TSR, consisting of teacher–student closeness and conflict. Teacher–student closeness is characterized by supportive relationships, mutual responsiveness, high positive affect, and emotional closeness. In contrast, teacher–student conflict reflects discordant and insensitive relationships with a high level of negative affect and hostility (O’Connor et al., 2012). Therefore, Students’ well-being, motivation, engagement, and achievement will depend on how teachers meet their basic needs (Ryan and Deci, 2017). On the other hand, dehumanization theory has opposite consequences, such as social ostracism, that generate leads to seeing oneself as an object, emotionally inert, cold, and rigid (Haslam et al., 2005; Andrighetto et al., 2017). According to the above information, our hypothesis is as follows:

H1: The TSR has an impact on the perception of organizational dehumanization. Specifically, the closer and warmer the relationship between teacher and student, the lower the Student’s perception of dehumanization.

The gender variable has been used in numerous studies as a moderating variable (Lietaert et al., 2015; He et al., 2019; Sladek et al., 2020). Several authors studied that in the case of the impact of TSR on Students’ learning behavior, it may be different for men and women (Hamre and Pianta, 2001; Roorda et al., 2011; Thornberg et al., 2020). According to the gender role socialization perspective, girls may benefit more from close relationships with the teacher because closeness is consistent with the greater intimacy and affiliation in social relationships expected of them (Maccoby, 1999). In addition, they may be more blocked by conflictive TSR because conflict-related behaviors, such as aggression and dominance, are generally less accepted for girls than for boys (Ewing and Taylor, 2009). In this study, gender is theorized to be an influencing factor between the relationship within ingroup–outgroup and dehumanization of ingroup. Therefore, the second hypothesis is:

H2: Gender (male/female) will moderate the relationship between the TSR and organizational dehumanization.

Status has been studied under the theory of infrahumanization and organizational dehumanization with different results. Based on the infrahumanization theory, Brauer (2001) and Rodríguez-Pérez et al. (2011) demonstrated that the socioeconomic level is not decisive for the attribution of more or less humanity to the outgroup.

On the other hand, Turner and Reynolds’ (2010) research on social identity asserted that when status differences are perceived as legitimate, members of low-status groups show outgroup favoritism whereas members of high-status groups show ingroup favoritism. Moreover, Russo and Mosso (2019) analyzed dehumanization under the theory of legitimacy of status and corroborated that ingroup does not dehumanize the outgroup when the outgroup is perceived as legitimately superior by the ingroup. Therefore, the aim of this study is to demonstrate whether the ingroup is perceived as dehumanizing depending on the status and the relationship with the outgroup.

H3: Status (low/high) moderates the relationship with the outgroup and the organizational dehumanization perception.

## Materials and Methods

### Participants

A total of 528 participants completed the study; 66% (350/528) of them were women. Participants were professors (26%) and students (74%) from different faculties (Humanities and Arts, Social and Legal Sciences, Health Sciences, Sciences and Engineering, and Architecture) ranging in age from 18 to 68 (mean 30.18, SD 14.23).

### Instruments

#### Organizational Dehumanization

Organizational dehumanization was assessed using the Organizational Dehumanization Scale of Caesens et al. (2017), adapted to Spanish by Ariño-Mateo et al. (in press). It consists of 10 items that are answered on a Likert scale from 1 (“total disagreement”) to 7 (“total agreement”). The example item is “My faculty treats me as a tool for their own success.” Cronbach’s alpha in this study was 0.935 and McDonald’s omega was 0.938.

#### Relationship With the Outgroup

The relationship between professors and students was measured by means of an instrument composed of four items developed for this study. Participants responded on a Likert scale from 1 (“total disagreement”) to 7 (“total agreement”). The questions were oriented according to the responding group, students (low status) and teachers (high status). An example is “I feel valued by the teachers who teach me” in the case of students and “I perceive my relationship with the class group I teach as good” for teachers. Cronbach’s alpha in this study was 0.821, and McDonald’s omega was 0.879.

A confirmatory factor analysis was performed for each instrument using the JASP software. Table 1 shows the results, which indicate that each instrument is correctly adjusted.

**TABLE 1:** *CFA* results of the scales used in the study.

	χ^2^	*df*	χ^2^/*df*	SRMR	RMSEA	CFI	TLI
Organizational dehumanization	86.192	25	3.44	0.06	0.03	0.98	0.97
Relationship with the outgroup	62.60	31.3	2.77	0.03	0.24	0.94	0.84

*N = 528.*

*SRMR, standardized root-mean-square residual; RMSEA, root-mean-square error of approximation; CFI, comparative fit index; TLI, Tucker-Lewis Index.*

### Design and Procedure

Participants were contacted at the institutional email and were voluntarily invited to complete the survey by clicking on the link. The survey was prepared using Google Forms Instructions for completing the questionnaires appeared on the survey home page and in the email. The questionnaire consisted of two sections. The first section asked for sociodemographic data and characteristics of the participants, and the second section focused on the study variables.

A 2-week period was provided for the completion of the questionnaire, which could be completed in 10 min. The moderating model was calculated using PROCESS, a mediation and moderation software package (Hayes, 2012).

### Analysis

**FIGURE 1:**
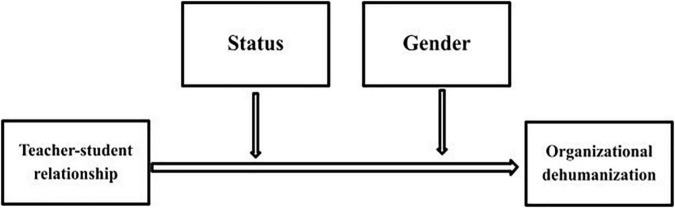
Model of moderation between TSR, status, gender, and organizational dehumanization.

Descriptive, correlational, and moderation analyses were performed. Relationship with others was considered as an independent variable, organizational dehumanization as a dependent, and status and gender as moderating variables. The moderator model of analysis measured: (b1) the effect of the relationship with the outgroup on organizational dehumanization; (b2) the effect of participant status (high/low) on organizational dehumanization; (b3) the effect of gender on the organizational dehumanization; (b4) the interaction effect of relationship with the outgroup and participant status on the organizational dehumanization; and (b5) the interaction effect of relationship with the outgroup and gender on organizational dehumanization. Figure 1 shows the proposed model.

## Results

### Results of Psychometric Adjustment of the Instruments

Confirmatory factor analysis was applied to analyze the construct validity of the instruments used (Table 1). In the case of the organization dehumanization scale, the indicators show that the model fits well, X2 = 86.19, df = 25, X2/df = 3.44 (< 3), SRMR = 0.03 (< 0.08), RMSEA = 0.06 (< 0.08), CFI = 0.98 (> 0.90), and TLI = 0.97 meet the criteria described. The relationship with the outgroup was also correctly adjusted (X2 = 62.60; df = 2 and X2/df = 2.57; SRMR = 0.03; RMSEA = 0.24; CFI = 0.94; TLI = 0.84).

Table 2 shows the means, SD, and correlations of the study variables. The results indicated a mean of 4.47 (SD = 1.51) for outgroup relationships and 4.25 (SD = 1.61) for organizational dehumanization. Both scales have the same range of 1 as minimum and 7 as maximum. The *t*-test was calculated to measure the correlation between gender and status with the outgroup relationship. In relation to the TSR, there is a significant difference in gender (*t* = 2.481, *p* < 0.01) and status (*t* = 16.234, *p* < 0.01 In the case of organizational dehumanization, the Pearson test was used, where the results indicate a negative correlation (*r* = −0.405, *p* < 0.01). Thus, empirical support was obtained for Hypothesis 1, that is, the TSR is negatively related to the perception of organizational dehumanization.

**TABLE 2:** Descriptive statistics of relationship with the outgroup and organizational dehumanization and correlation matrix between the study variables of the total sample.

	Min	Max	Mean	SD	1	2
Relationship with the outgroup	1	7	4.47	1.51	(0.870; 0.880) 1	−0.405**
Organizational dehumanization	1	7	4.25	1.61		(0.936; 0.937) 1

*N = 528.*

**p < .01.

### Descriptive Statistics, Correlations, and Moderation Analysis

Table 3 presents the empirical contrast of Hypotheses 1, 2, and 3. The first hypothesis is confirmed by the results since there is a significant effect on Students’ dehumanization according to the relationship they have with teachers (*p* < 0.001). That is, the better the relationship, the lower the perceived dehumanization. In relation to Hypotheses 2 and 3, these are also confirmed. The results show that the relationships between the outgroup and organizational dehumanization are moderated by status (*p* < 0.001) and gender (*p* < 0.001).

**TABLE 3:** Moderation model using status and gender between relationship with the outgroup and organizational dehumanization.

Variables	Coefficient	T	*P*	LLCI	ULCI
Constant	4.97	11.43	0.000	4.12	5.83
Gender	1.52	3.75	0.000	0.73	2.32
Rel	−0.25	−3.31	0.001	−0.39	−0.10
RelXGender	−0.25	−2.94	0.004	−0.42	−0.08
Status	2.86	3.28	0.001	1.15	4.57
RelXStatus	−0.56	−3.74	0.000	−0.86	−0.27

*95% level of confidence. Rel, Relationship with the outgroup. The reference group is men.*

**FIGURE 2:**
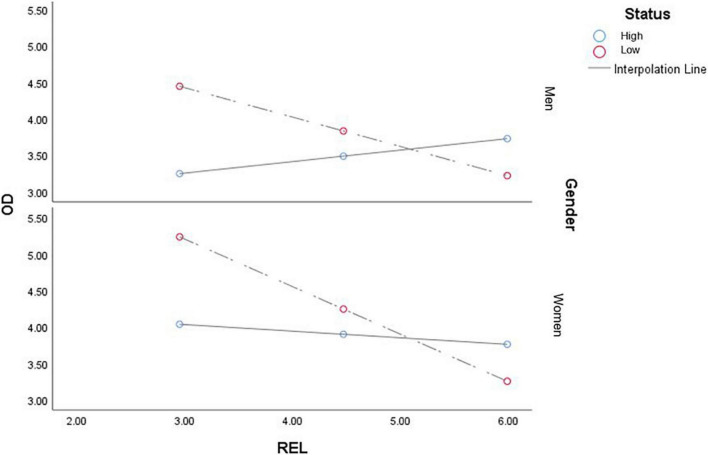
Conditional effect that the relationship with the outgroup has on the perception of organizational dehumanization by gender and status.

Figure 2 shows that the effect of the relationship with the outgroup (teachers-students) on organizational dehumanization is affected by status, specifically low-status participants (students). That is, the better the relationship with teachers, the fewer students perceive themselves to be dehumanized by the organization, regardless of gender: men (−0.403, *p* < 0.01) and women (−0.652, *p* < 0.01). However, when the status is high, this effect is not significant for both men (1.0952, *p* > 0.01) and women (−0.6195, *p* > 0.01), so we cannot confirm that teachers are dehumanized by the outgroup. Furthermore, gender plays an important role, since when the participant group is in the low status, the effect of the relationship with high status on the perception of dehumanization affects women more than men (1.52, *p* < 0.01).

## Discussion

This work represents an advance in the understanding of the variables and contexts of organizational dehumanization, such as gender and educational environment, which have not been previously investigated. For this study, three hypotheses were tested and accepted.

In regard to the gender variable, women have a higher perception of organizational dehumanization than men when they have the same relationship with the high-status outgroup. This result goes on the same line with the socialization perspective which corroborates that a poor relationship with the outgroup affects women more significantly than men (Maccoby, 1999; Thornberg et al., 2020).

Moreover, these findings have a great effect in practice for several reasons. First, this study reveals that female employees who do not have a close or good relationship with their leader are more likely to be organizationally dehumanized. Second, individuals with high perceived dehumanization tend to have consequences such as psychosomatic stress, emotional exhaustion, job satisfaction, and organizational citizenship behaviors (Caesens et al., 2017; Sarwar, 2020; Arriagada-Venegas et al., 2021). Therefore, the contribution of this research to gender issues is interesting, and future research is needed on how the aforementioned effects are influenced by gender.

Second, this research also contributes to the understanding of how status operates in organizational dehumanization. As we have previously commented, there is research that claims that status does not influence the attribution of greater or lesser humanity to the outgroup (Rodríguez-Pérez et al., 2011). Nevertheless, more recent studies confirmed the importance of the legitimacy of status and how the outgroup was less infrahumanized when it was legitimately perceived by the ingroup (Russo and Mosso, 2019). In this research, we have gone a step further and demonstrated that status affects our own perception of dehumanization and that it also moderates the relationship between the outgroup and the attribution of humanization.

Finally, the most representative of this article is how gender and status affect organizational dehumanization, such that women with lower organizational rank perceived themselves to be more dehumanized than men when the relationship with the high-status group is not close or just good. On the other hand, the high-status group, regardless of gender, has a less dehumanizing effect, meaning that women and men are independently perceived as less dehumanized when they are in a high-status role.

Consequently, women with low status have the worst perceptions of dehumanization. This last finding is interesting because it provides a hierarchy between the preponderance of gender and status.

Regarding the applied implications of the results of this study, there is at least one line of potentially useful practical suggestions for educational establishments and organizations in various settings. First, teachers or high-status people should be aware of how their position affects the perception of dehumanization in others, especially in low-status women outgroup. Because in this way corrective measures can be taken to reduce the negative effects of organizational dehumanization on those of low status, as well as in the case of women. Furthermore, the organization should take special care of female employees, as they are generally much more susceptible to being organizationally dehumanized, and should consider that for this gender is crucial to have a close and good relationship in the organization.

For future research, it would be very interesting to analyze the effects that organizational dehumanization has across different types of organizations according to hierarchy because this research looked for differences in status in the university context where hierarchy is marked. It could be possible that organizations with flat hierarchies have low differences in the perception of organizational dehumanization of their employees (in low and high status).

Despite the various contributions provided, it is necessary to point out the limitations of this study that should be considered for future research. This study had a cross-sectional approach, and it is recommended to carry out experimental or longitudinal studies to rectify the results presented over a long period of time.

## Conclusion

This research contributes to the understanding of organizational dehumanization theory and other variables such as gender, status, type of relationship within groups, and the organizational contexts, specifically the university context. The main conclusion is the importance for women to have a close relationship with the professor to avoid the perception of being dehumanized in the university. Further research is needed to investigate how organizational dehumanization is different by gender and what other variables affect dehumanization.

## Data Availability Statement

The raw data supporting the conclusions of this article will be made available by the authors, without undue reservation.

## Ethics Statement

The studies involving human participants were reviewed and approved by the CEIBA 2020-0411. Written informed consent to participate in this study was provided by the participants’ legal guardian/next of kin.

## Author Contributions

MA-V and EA-M analyzed the data and drafted the initial version of the article. All authors contributed to the conception and design of the study, data collection, results’ interpretation, and approval of the final version.

## Conflict of Interest

The authors declare that the research was conducted in the absence of any commercial or financial relationships that could be construed as a potential conflict of interest.

## Publisher’s Note

All claims expressed in this article are solely those of the authors and do not necessarily represent those of their affiliated organizations, or those of the publisher, the editors and the reviewers. Any product that may be evaluated in this article, or claim that may be made by its manufacturer, is not guaranteed or endorsed by the publisher.
